# Development of an S-1 dosage formula based on renal function by a prospective pharmacokinetic study

**DOI:** 10.1007/s10120-015-0536-6

**Published:** 2015-08-25

**Authors:** Eisuke Booka, Chiyo K. Imamura, Hiroya Takeuchi, Yasuo Hamamoto, Daisuke Gomi, Takuro Mizukami, Takashi Ichiyama, Kazunari Tateishi, Tsunehiro Takahashi, Hirofumi Kawakubo, Kenzo Soejima, Narikazu Boku, Yusuke Tanigawara, Yuko Kitagawa

**Affiliations:** Department of Surgery, Keio University School of Medicine, 35 Shinanomachi, Shinjuku-ku, Tokyo, 160-8582 Japan; Department of Clinical Pharmacokinetics and Pharmacodynamics, School of Medicine, Keio University, 35 Shinanomachi, Shinjuku-ku, Tokyo, 160-8582 Japan; Keio Cancer Center, Keio University Hospital, 35 Shinanomachi, Shinjuku-ku, Tokyo, 160-8582 Japan; Department of Comprehensive Cancer Therapy, Shinshu University School of Medicine, 3-1-1 Asahi, Matsumoto, Nagano 390-8621 Japan; Department of Clinical Oncology, St. Marianna University School of Medicine, 2-16-1 Sugao, Miyamae-ku, Kawasaki, Kanagawa 216-8511 Japan; First Department of Internal Medicine, Shinshu University School of Medicine, 3-1-1 Asahi, Matsumoto, Nagano 390-8621 Japan; Division of Pulmonary Medicine, Department of Medicine, Keio University School of Medicine, 35 Shinanomachi, Shinjuku-ku, Tokyo, 160-8582 Japan

**Keywords:** Creatinine clearance, Dosage formula, Population pharmacokinetics, Renal function, S-1

## Abstract

**Background:**

S-1 is an oral anticancer drug, containing tegafur (a prodrug of 5-fluorouracil, 5-FU), 5-chloro-2,4-dihydroxypyridine, and potassium oxonate. As renal dysfunction is known to increase exposure of 5-FU following S-1 administration, the incidence of severe adverse reactions is increased in patients with impaired renal function. However, no reliable information on its dose modification for patients with renal dysfunction has been provided.

**Methods:**

We conducted a prospective pharmacokinetic study to develop an S-1 dosage formula based on renal function. Sixteen cancer patients with various degrees of renal function received a single dose of S-1 at 40 mg/m^2^. A series of blood samples were collected at predefined times within 24 h to assess the plasma concentration profiles of 5-FU, 5-chloro-2,4-dihydroxypyridine, and tegafur. A mathematical model for the relationship between renal function and exposure of 5-FU was constructed by a population pharmacokinetic analysis.

**Results:**

The clearance of 5-FU following S-1 administration was related to body surface area and creatinine clearance in the range 15.9–108.8 mL/min as estimated by the Cockcroft–Gault equation. The S-1 dosage formula was derived as follows:

$${\text{dose}} = {\text{target AUC}} \times \left( {21.9 + 0.375 \times {\text{CLcr}}} \right) \times {\text{BSA}},$$

where AUC is the area under the concentration–time curve, CLcr is creatinine clearance, and BSA is body surface area. The recommended daily doses of S-1 in Asia and Europe were also proposed as nomograms according to exposure matching to the previously reported area under the concentration–time curve of 5-FU, which confirmed the efficacy and toxicity in pivotal registration studies.

**Conclusions:**

We have developed a novel formula for determining the S-1 dosage on the basis of renal function. Further validation is needed to confirm the formula for practical application.

**Electronic supplementary material:**

The online version of this article (doi:10.1007/s10120-015-0536-6) contains supplementary material, which is available to authorized users.

## Introduction

After entering the body, a drug is eliminated by metabolism and/or excretion. Although elimination can occur via several routes, most drugs are cleared by metabolism in the liver and/or elimination through the kidney. For a drug eliminated primarily via renal excretory mechanisms, impaired renal function alters its pharmacokinetics to an extent that its dosage needs to be changed from that used in patients with normal renal function. The US Food and Drug Administration [1] and the European Medicines Agency [2] recommend that the pharmacokinetics of a new drug be assessed in patients with impaired renal function during the development phase and that rational dosing recommendations be provided. However, there is no similar guidance in Japan; thus, dose modification for patients with renal dysfunction is based on data from the USA and Europe.

**Fig. 1:**
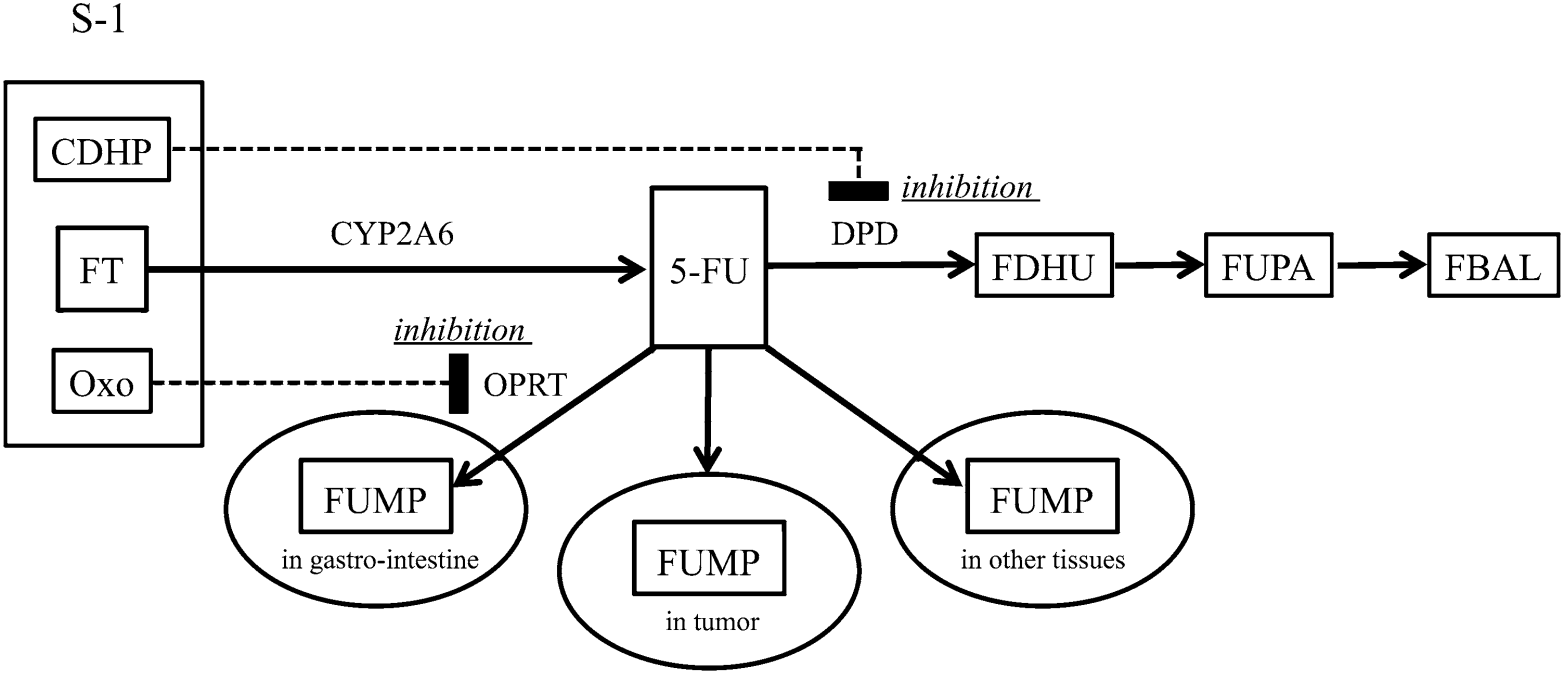
Biochemical action of S-1. *CDHP* 5-chloro-2,4-dihydroxypyridine, *CYP2A6* cytochrome P450 2A6, *DPD* dihydropyrimidine dehydrogenase, *FBAL* α-fluoro-β-alanine, *FDHU* fluorodihydrouracil, *FT* tegafur, *5-FU* 5-fluorouracil, *FUMP* fluorouridine monophosphate, *FUPA* α-fluoro-β-ureidopropionic acid, *OPRT* orotate phosphoribosyltransferase, *Oxo* potassium oxonate

Since Heidelberger et al. [3] reported 5-fluorouracil (5-FU) in 1957, 5-FU has been a key drug for gastrointestinal tumors. An oral drug S-1 was first approved in Japan in 1999 and is widely used in Asia and Europe (TS-1^®^, Taiho Pharmaceutical, Tokyo, Japan, in Asia; Teysuno^®^, Nordic Group, Hoofddrop, The Netherlands, in Europe). S-1 contains tegafur, which is a prodrug of 5-FU, 5-chloro-2,4-dihydroxypyridine (CDHP), which inhibits the rate-limiting enzyme (dihydropyrimidine dehydrogenase) of 5-FU catabolism to maintain a high concentration of 5-FU, and potassium oxonate, which is specifically distributed in the epithelium of the intestine and inhibits the phosphorylation of 5-FU to reduce gastrointestinal toxicity [4–6] (Fig. 1). The approved dosage and administration of S-1 are 80 mg/m^2^/day as tegafur for 4 weeks followed by 2 weeks of rest in Asia [7, 8], and 50 mg/m^2^/day as tegafur for 3 weeks followed by 1 week of rest in combination with cisplatin at 75 mg/m^2^ once every 4 weeks in Europe [9]. S-1 is a standard adjuvant treatment for East Asian patients who have undergone a D2 dissection for locally advanced gastric cancer [10] and it is noninferior to 5-FU for patients with metastatic gastric cancer [11]. S-1 in combination with cisplatin is a standard first-line treatment for patients with advanced gastric cancer [12].

Since more than 50 % of CDHP is excreted in urine [13], renal dysfunction increases exposure of CDHP, and results in a sustained high concentration of 5-FU [14]. The results of a postmarketing survey of S-1 administered at 80 mg/m^2^/day as tegafur revealed that the incidence of grade 3 or grade 4 adverse reactions increased in patients with impaired renal function. S-1 is contraindicated in patients with severe renal dysfunction, but no information on dose modification for patients with mild and moderate renal dysfunction is provided on the Asian package inserts [7, 8].

We therefore attempted to develop an S-1 dosage formula based on renal function to determine the recommended dose for patients with impaired renal function. A prospective pharmacokinetic study was conducted to assess the plasma concentration profiles of 5-FU, CDHP, and tegafur in patients with various degrees of renal function. A mathematical model for the relationship between renal function and exposure of 5-FU was constructed by a population pharmacokinetic (PPK) analysis.

## Patients and methods

### Patient eligibility

The eligibility criteria of this study were as follows: histologically confirmed solid tumor in patients for whom administration of S-1 was planned; creatinine clearance (CLcr) estimated by the Cockcroft–Gault equation [15] of 15 mL/min or greater; age 20 years or older; Eastern Cooperative Oncology Group performance status 0–2; adequate organ function except renal function (white blood cell count 3000–12,000/mm^3^; neutrophil count 1500/mm^3^ or greater; platelet count 100,000/mm^3^ or greater; total bilirubin concentration 2.0 mg/dL or lower; aspartate transaminase and alanine transaminase concentration 100 U/L or lower); ability to take medications orally; and no previous administration of S-1 within 14 days if used.

The study was performed as a project of the Promotion Plan for the Platform of Human Resource Development for Cancer by the Ministry of Education, Culture, Sports, Science and Technology in Japan. The study protocol was approved by each participating institution’s institutional review board and was registered in the University Hospital Medical Information Network Clinical Trials Registry under the number UMIN000011708. The study procedures were in accordance with the ethical standards of the Declaration of Helsinki. Written informed consent was obtained from all patients before their enrollment in the study.

### Study design

This prospective pharmacokinetic study in cancer patients with various degrees of renal function was conducted at three institutions in Japan. The primary endpoint was to assess the pharmacokinetic profiles of 5-FU, CDHP, and tegafur after a single dose of S-1 in patients with various degrees of renal function. The secondary endpoint was to evaluate the toxicity within 24 h of S-1 administration in the patients. US National Cancer Institute Common Terminology Criteria for Adverse Events version 4.0 was used for the toxicity assessment [16]. The study duration was 24 h after administration of S-1.

### Drug formulation and administration

S-1 (TS-1^®^) orally disintegrating tablets containing tegafur, CDHP, and potassium oxonate in a molar ratio of 1:0.4:1 were administered to the patients. Two dosages of tegafur were used: 20 and 25 mg.

The enrolled patients received a single dose of S-1 at 40 mg/m^2^ as tegafur, which is the approved regular single dose and half of the daily dose in Asia [7, 8, 10]. S-1 was taken within 30 min after breakfast at a dose of 40 mg as tegafur (two tablets each containing 20 mg) for patients with a body surface area (BSA) of less than 1.25 m^2^, 50 mg as tegafur (two tablets each containing 25 mg) for those with 1.25 m^2^ ≤ BSA < 1.50 m^2^, and 60 mg as tegafur (three tablets each containing 20 mg) for those with BSA ≥ 1.50 m^2^.

### Estimation of renal function

To assess the impact of renal function on the pharmacokinetics of CDHP and 5-FU, two commonly used serum-creatinine-based equations, the Cockcroft–Gault equation and the Japanese glomerular filtration rate (GFR) equation [17], were used to estimate renal function.

### Pharmacokinetic sample acquisition and handling

Blood samples were obtained before and 1, 2, 4, 7, 12, and 24 h after administration. Peripheral blood samples were drawn into heparinized tubes at a volume of 3 mL at each sampling time and were centrifuged at 3000 rpm for 10 min at room temperature. The resulting plasma was frozen and stored at −20 °C until analysis.

### Determination of the plasma concentrations of 5-FU, CDHP, and tegafur

The plasma concentrations of 5-FU, CDHP, and tegafur were determined with an ultraperformance liquid chromatography (UPLC)–tandem mass spectrometry method developed specifically for this study by modification of a previously reported method [18]. 5-FU, tegafur, and 5-bromouracil, an internal standard, were purchased from Sigma-Aldrich (St Louis, MO, USA). CDHP was obtained from Toronto Research Chemicals (North York, ON, Canada).

Patient plasma (100 µL) or an equivalent volume of plasma that contained known concentrations of 5-FU, CDHP, and tegafur was mixed with 250 µL of the internal standard solution (5-bromouracil at 100 ng/mL in acetonitrile). The mixture was vortexed and centrifuged at 13,000 rpm for 5 min. The resulting supernatant was transferred to a glass tube and evaporated to dryness under air at 40 °C. The dried residue was reconstituted in 75 µL of 0.1 % formic acid–acetonitrile (90:10, v/v) and transferred to an autosampler vial tube.

The UPLC-tandem mass spectrometry system was equipped with an Acquity UPLC system and a Xevo TQ mass spectrometer (Waters, Milford, MA, USA). Chromatographic separations were obtained under gradient conditions with an ACQUITY UPLC HSS T3 column (100 mm × 2.1-mm inner diameter, 1.8-µm particle size; Waters). The mobile phase consisted of eluent A (0.1 % formic acid) and eluent B (acetonitrile). The flow rate was 0.3 mL/min, and the gradient was as follows: 8 % eluent B for 2 min, 95 % eluent B at 4.5 min, and 8 % eluent B at 6.6 min. The total run time was 9 min per sample. The column temperature was 40 °C, the sample temperature was 10 °C, and the injection volume was 5 µL. Under these conditions, the retention times for 5-FU, CDHP, tegafur, and 5-bromouracil (internal standard) were 1.07, 2.59, 3.54, and 1.54 min, respectively.

The mass spectrometer was run in negative electrospray ionization mode for 5-FU and in the positive mode for CDHP and tegafur. The source conditions were as follows: capillary voltage, 3 kV; cone voltage, 30 V; and desolvation temperature, 400 °C. A collision gas flow rate of 0.28 mL/min and collision energy of 5 keV were used for the creation of daughter ions. The multiple reaction monitoring mode detected the following transitions: 129.1 → 41.9 and 189.0 → 41.9 for 5-FU and 5-bromouracil, respectively, in the negative mode and 146.1 → 72.9, 201.2 → 131.0, and 191.1 → 117.8 for CDHP, tegafur, and 5-bromouracil, respectively, in the positive mode. The chromatographic data were acquired and analyzed with MassLynx equipped with QuanLynx (Waters).

The concentrations of 5-FU, CDHP, and tegafur in patient plasma samples were calculated by determining the ratios of the areas of 5-FU, CDHP, and tegafur in each sample to the area of the internal standard in that sample and by comparing these ratios to the standard curve prepared on the same day as the samples. The concentration ranges of the standard curves were 5–500 ng/mL for 5-FU and CDHP and 25–2500 ng/mL for tegafur. The interday and intraday variabilities in precision (expressed as the coefficient of variation) for all compounds ranged from 0.3 to 5.8 % and from 4.7 to 8.9 %, respectively. The average accuracies for the compounds ranged from 100.7 to 107.6 %.

### PPK analysis

The PPK analysis was performed with NONMEM (version 7.2; ICON Development Solutions, Dublin, Ireland) and GNU Fortran compiler version 4.6.0 on Microsoft Windows 7. The basic pharmacokinetic parameters were clearance, volume of distribution, and the first-order absorption rate constant (*K*_a_). As all doses were given by oral administration, clearance and the volume of distribution were interpreted as the ratio of clearance to bioavailability and the ratio of the volume of distribution to bioavailability, respectively.

As a pharmacokinetic structural model of CDHP, 5-FU, and tegafur, a linear one-compartment model with first-order absorption (subroutines ADVAN2 and TRANS2) without the absorption lag time was used because it was reported that a one-compartment model was more suitable than a two-compartment model and the introduction of the absorption lag time did not improve the model fitting [19].

The interindividual variability was assumed to obey a log-normal distribution and is described for each parameter as follows:

$$\theta_{j} = \, \theta \, \times \, \exp \, \left( {\eta_{j} } \right),$$

where *η*_*j*_ is the random effect for individual *j*, *θ* is the population mean parameter, and *η* is a random variable with mean zero and variance *ω*^2^. Residual variability was described by a proportional error model as follows:

$$C_{i, \, j} = {\text{ Cpred}}_{i, \, j} \exp \, \left( {\varepsilon_{i, \, j} } \right),$$

where Cpred_*i, j*_ is the *i*th model-predicted concentration for patient *j*, *C*_*i*, *j*_ is the measured concentration, and *ε*_*i*, *j*_ denotes the residual intraindividual random error.

Demographic variables, such as renal function, patient age, sex, BSA, and history of gastrectomy, were examined to identify whether these variables could explain the observed substantial interindividual variability. Demographic variables were included one at a time by stepwise selection based on the likelihood ratio test. The minimum value of the NONMEM objective function was used as a statistic for choosing suitable models during the model-building process. The potentially significant covariates were identified as those factors that when added to the basic model individually resulted in a decrease in the objective function of 3.84 or more (*p* < 0.05).

To evaluate the validity and robustness of the PPK model obtained, a nonparametric bootstrap resampling method was conducted. One thousand bootstrap resampled data sets were generated, each containing the same number of patients as the original data set, and each of them was fitted individually to the final PPK model. Median values and 95 % confidence intervals for parameter estimates were compared with the parameter estimates obtained from the original data set. Furthermore, to check the suitability of the final model with respect to the observation, a visual predictive check was performed. On the basis of the final PPK model, 1000 plasma concentration profiles following the same dose were simulated without residual errors, and 95 % prediction intervals at each time point were plotted with the observed data.

### Statistical analysis

Statistical analysis was performed with IBM SPSS Statistics version 22 (IBM, Armonk, NY, USA). The relationships between the variables were analyzed by linear regression. A *p* value of less than 0.05 was considered statistically significant.

## Results

### Patient characteristics

Sixteen patients with CLcr in the range 15.9–108.8 mL/min were enrolled between December 2013 and November 2014 (Table 1). The patients were classified into four groups according to renal function as follows: four patients in the normal renal function group (CLcr ≥ 80 mL/min), five patients in the mild dysfunction group (CLcr = 60–79 mL/min), five patients in the moderate dysfunction group (CLcr = 30–59 mL/min), and two patients in the severe dysfunction group (CLcr < 30 mL/min).

**Table 1:** Patients’ characteristics

Characteristics	Renal function (CLcr, mL/min)				Total
	Normal (≥80)	Mild (60–79)	Moderate (30–59)	Severe (<30)	
No. of patients	4	5	5	2	16
CLcr (mL/min)					
Mean	97.3	67.1	48.0	22.6	63.1
Range	81.7–108.8	60.7–72.1	40.1–58.2	15.9–29.3	15.9–108.8
Serum creatinine (mg/dL)					
Mean	0.91	0.87	1.04	1.48	1.00
Range	0.68–1.39	0.48–1.25	0.61–1.28	1.30–1.66	0.48–1.66
Age (years)					
Median	55.5	65	76	71	65.5
Range	46–65	45–78	54–78	66–76	45–78
Weight (kg)					
Mean	73.6	55.4	54.4	36.6	57.3
Range	56.5–87.0	42.8–68.3	48.5–64.0	30.2–42.9	30.2–87.0
BSA (m^2^)					
Mean	1.874	1.589	1.596	1.298	1.626
Range	1.704–2.020	1.328–1.734	1.477–1.801	1.180–1.416	1.180–2.020
Sex					
Male	4	3	3	1	11
Female	0	2	2	1	5
PS					
0	4	4	4	0	12
1	0	0	1	1	2
2	0	1	0	1	2
Tumor type					
Stomach	3	3	5	0	11
Others	1	2	0	2	5
Gastrectomy					
Yes	2	3	4	0	9
No	2	2	1	2	7
S-1 dose (mg/m^2^)					
Mean	32.1	35.4	35.1	34.6	34.4
Range	29.7–35.2	32.6–37.7	33.3–39.6	33.9–35.3	29.7–39.6

*BSA* body surface area, *CLcr* creatinine clearance estimated by the Cockcroft–Gault equation, *PS* performance status

### Pharmacokinetics of 5-FU, CDHP, and tegafur and toxic effects

**Fig. 2:**
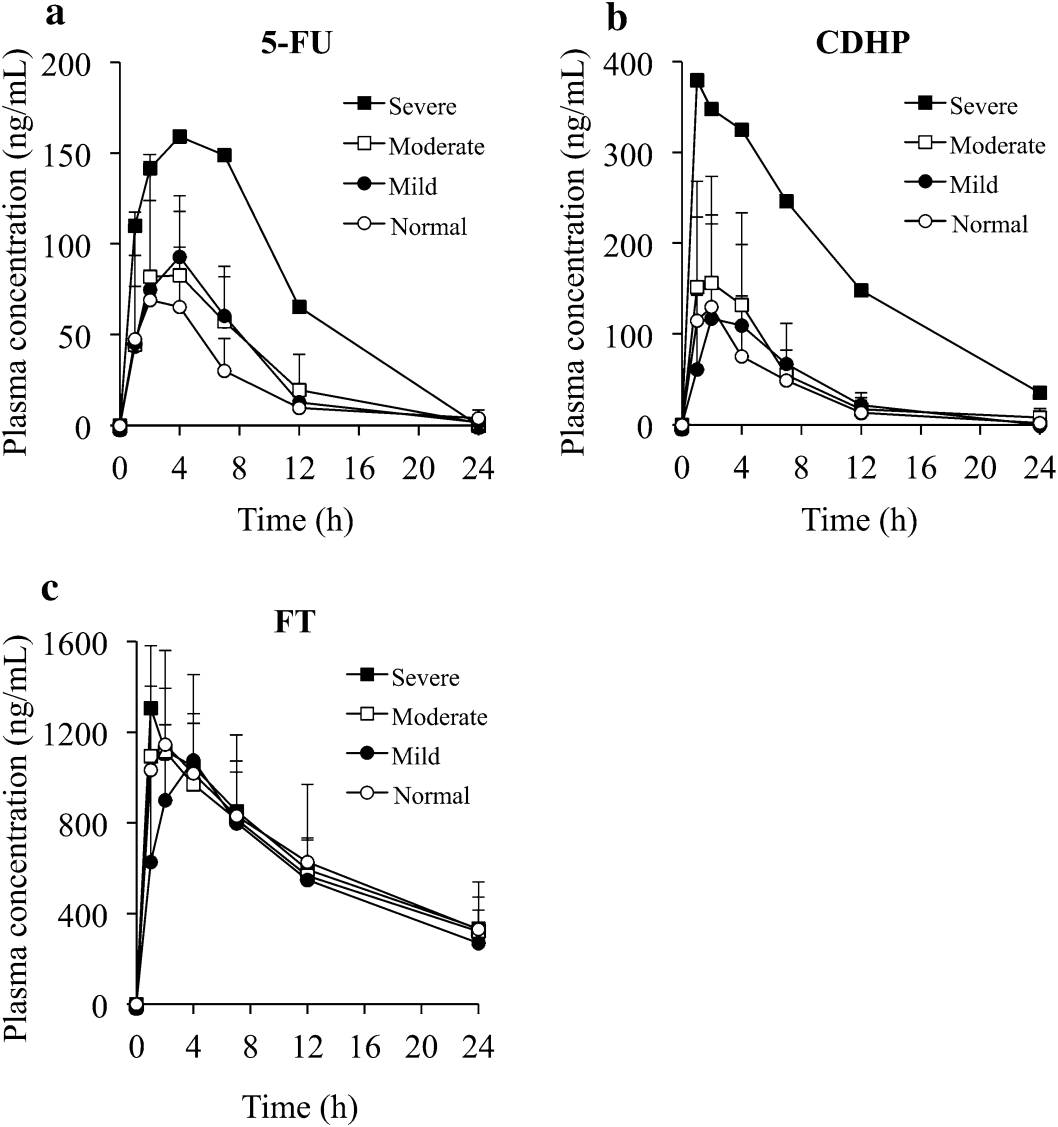
Mean (± standard deviation) plasma concentration–time profiles of 5-fluorouracil (*5-FU*; **a**), 5-chloro-2,4-dihydroxypyridine (*CDHP*; **b**), and tegafur (*FT*; **c**), according to renal function after a single dose of S-1

The plasma concentration–time profiles of 5-FU, CDHP, and tegafur according to renal function are shown in Fig. 2. Remarkable increases in the plasma concentrations of 5-FU and CDHP were observed in patients in the severe renal dysfunction group.

No adverse events were observed in any patients within 24 h of S-1 administration.

### Factors influencing the area under the concentration–time curve from 0 to 24 h of 5-FU

**Fig. 3:**
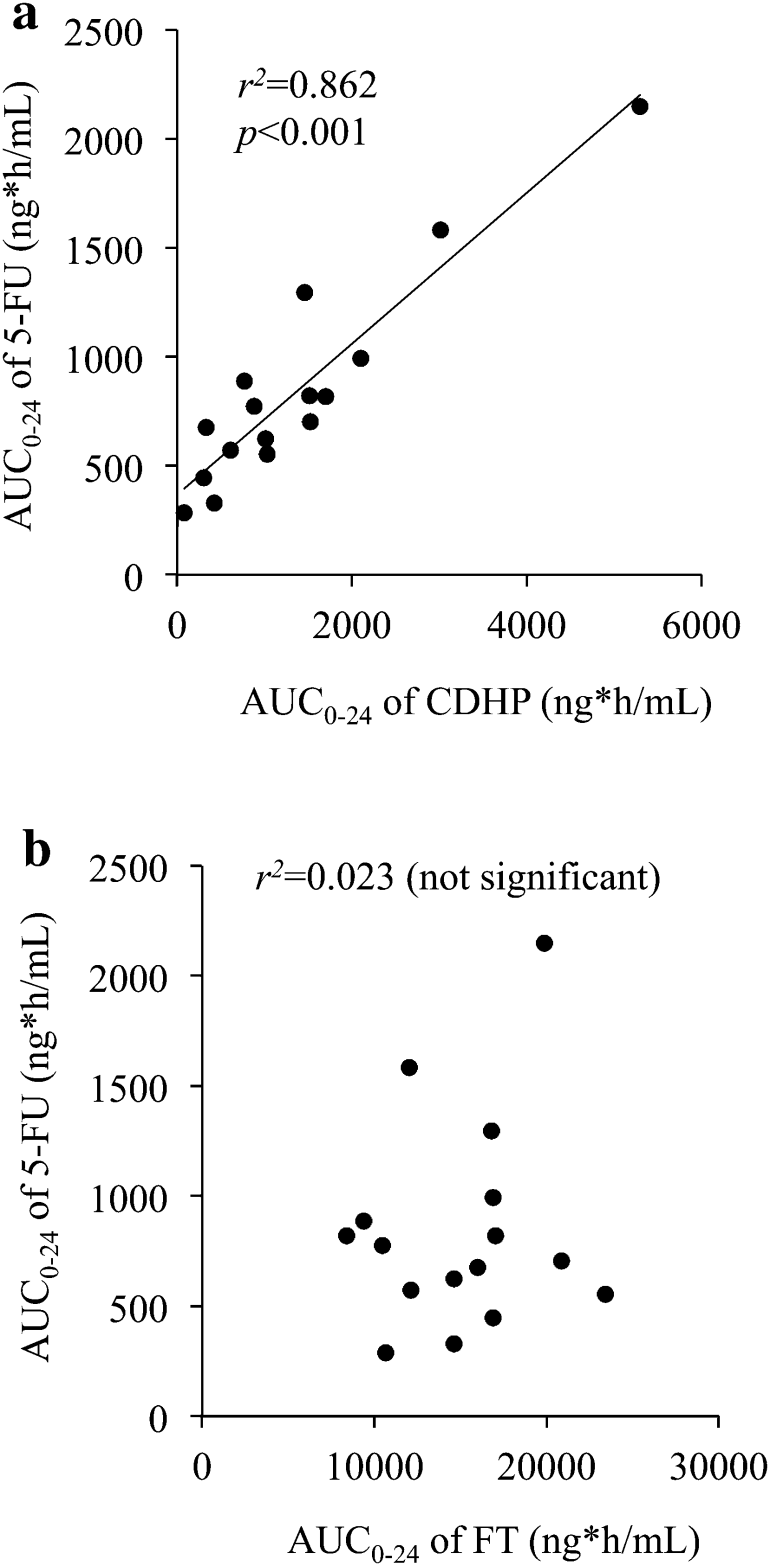
Correlation between the area under the concentration–time curve from 0 to 24 h (*AUC*_0-24_) of 5-fluorouracil (*5-FU*) and that of 5-chloro-2,4-dihydroxypyridine (*CDHP*) (**a**) or tegafur (*FT*) (**b**)

The area under the concentration–time curve (AUC) from 0 to 24 h (AUC_0–24_) of 5-FU, CDHP, and tegafur calculated with the linear trapezoidal rule in 16 patients was in the range 286.6–2149.9, 80.4–5295.9, and 8404.9–23403.8 ng h/mL, respectively. A moderately strong correlation was demonstrated between AUC_0–24_ of 5-FU and that of CDHP (*r*^2^ = 0.862, *p* < 0.001; Fig. 3a), whereas AUC_0–24_ of 5-FU was not correlated with that of tegafur (*r*^2^ = 0.023; Fig. 3b). This indicates that exposure of 5-FU is dependent on the exposure of CDHP but not on that of tegafur.

**Fig. 4:**
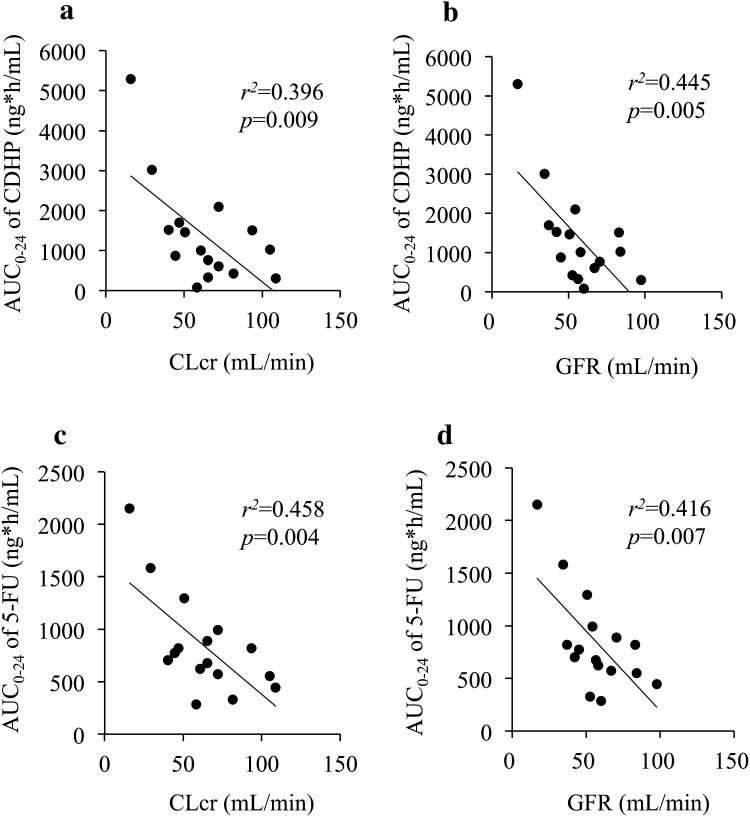
Correlation between the area under the concentration–time curve from 0 to 24 h (AUC_0–24_) of 5-chloro-2,4-dihydroxypyridine (CDHP) and creatinine clearance (CLcr) (**a**) or glomerular filtration rate (GFR) (**b**). Correlation between AUC_0–24_ of 5-fluorouracil (5-FU) and CLcr (**c**) or GFR (**d**). CLcr and GFR were estimated by the Cockcroft–Gault equation and the Japanese GFR equation, respectively

AUC_0–24_ of CDHP correlated moderately with CLcr (*r*^2^ = 0.396, *p* < 0.01; Fig. 4a), and with GFR (*r*^2^ = 0.445, *p* < 0.01; Fig. 4b). In addition, AUC_0–24_ of 5-FU correlated moderately with CLcr (*r*^2^ = 0.458, *p* < 0.01; Fig. 4c) and with GFR (*r*^2^ = 0.416, *p* < 0.01; Fig. 4d). Both CLcr and GFR could be considered as appropriate measures of renal function, which influenced exposure of both CDHP and 5-FU. We selected CLcr estimated by the Cockcroft–Gault equation as the value of renal function to simulate and predict the pharmacokinetics of 5-FU by PPK analysis because previous reports [14, 20, 21] and the summary of product characteristics in Europe [9] showed and discussed the impact of renal function on S-1 pharmacokinetics with CLcr, but not GFR, as a measure.

### PPK analysis of CDHP, 5-FU, and tegafur

The patients’ age, sex, and history of gastrectomy did not have substantial relationships with the individual clearance, volume of distribution, and *K*_a_ values of CDHP and 5-FU in the PPK analysis. The individual clearance values of CDHP and 5-FU were related to CLcr and BSA. In addition, the individual volume of distribution values of CDHP and 5-FU were proportional to the BSA. The estimated population mean and variance of the pharmacokinetic parameters of CDHP and 5-FU are shown in Table 2.

**Table 2:** Estimated parameters of the population pharmacokinetic model

Compound	Parameter	Population mean	Interindividual variability (%)
5-FU	*K*_a_ (1/h)	0.551	202
	CL/*F* (L/h/m^2^)	(21.9 + 0.375 × CLcr) × BSA	27.5
	*V*/*F* (L/m^2^)	362 × BSA	87.7
	Residual variability (%)	37.9	
CDHP	*K*_a_ (1/h)	1.04	167
	CL/*F* (L/h/m^2^)	(3.77 + 0.403 × CLcr) × BSA	49.9
	*V*/*F* (L/m^2^)	200 × BSA	79.0
	Residual variability (%)	33.3	
Tegafur	*K*_a_ (1/h)	1.46	121
	CL/*F* (L/h/m^2^)	2.89	41.8
	*V*/*F* (L/m^2^)	44.9	23.6
	Residual variability (%)	12.7	

*BSA* body surface area, *CDHP* 5-chloro-2,4-dihydroxypyridine, *CL* clearance, *CLcr* creatinine clearance estimated by the Cockcroft–Gault equation, *F* bioavailability, *5-FU* fluorouracil, *K*_*a*_ first-order absorption rate constant, *V* volume of distribution

The patients’ age, sex, history of gastrectomy, renal function, and BSA did not affect the clearance, volume of distribution, and *K*_a_ of tegafur in the PPK analysis. The estimated population mean and variance of the pharmacokinetic parameters of tegafur are also shown in Table 2.

### Model validation

The results of the visual predictive check and bootstrap validation are presented in Table S1 and Fig. S1. In the bootstrap resampling, more than 99 % of the generated data sets successfully converged. The median values for bootstrap simulation were very consistent with the final parameter estimates obtained with the original data set. These diagnostics and the validation indicated that the final model is robust and reliable for describing the pharmacokinetics of S-1 in patients with various degrees of renal function.

### Development of an S-1 dosage formula

The PPK parameters obtained were used to develop an S-1 dosage formula. The 5-FU clearance related to CLcr and BSA was described by the following equation (Table 2):

1 $${\text{CL}}/F \, = \, (21.9 \, + \, 0.375 \times {\text{CLcr}}) \times {\text{BSA}}$$

where CL is clearance and *F* is bioavailability. As AUC = F × dose/CL, Eq. 1 was rearranged to

2 $${\text{Dose/AUC}} = \, (21.9 \, + \, 0.375 \times {\text{CLcr}}) \times {\text{BSA}}$$

Equation 2 was further rearranged to provide the recommend S-1 dose as follows:

3 $${\text{Dose as FT }} = {\text{ target AUC of 5-FU}}\times (21.9 \, + \, 0.375 \times {\text{CLcr}}) \times {\text{BSA}}$$

**Fig. 5:**
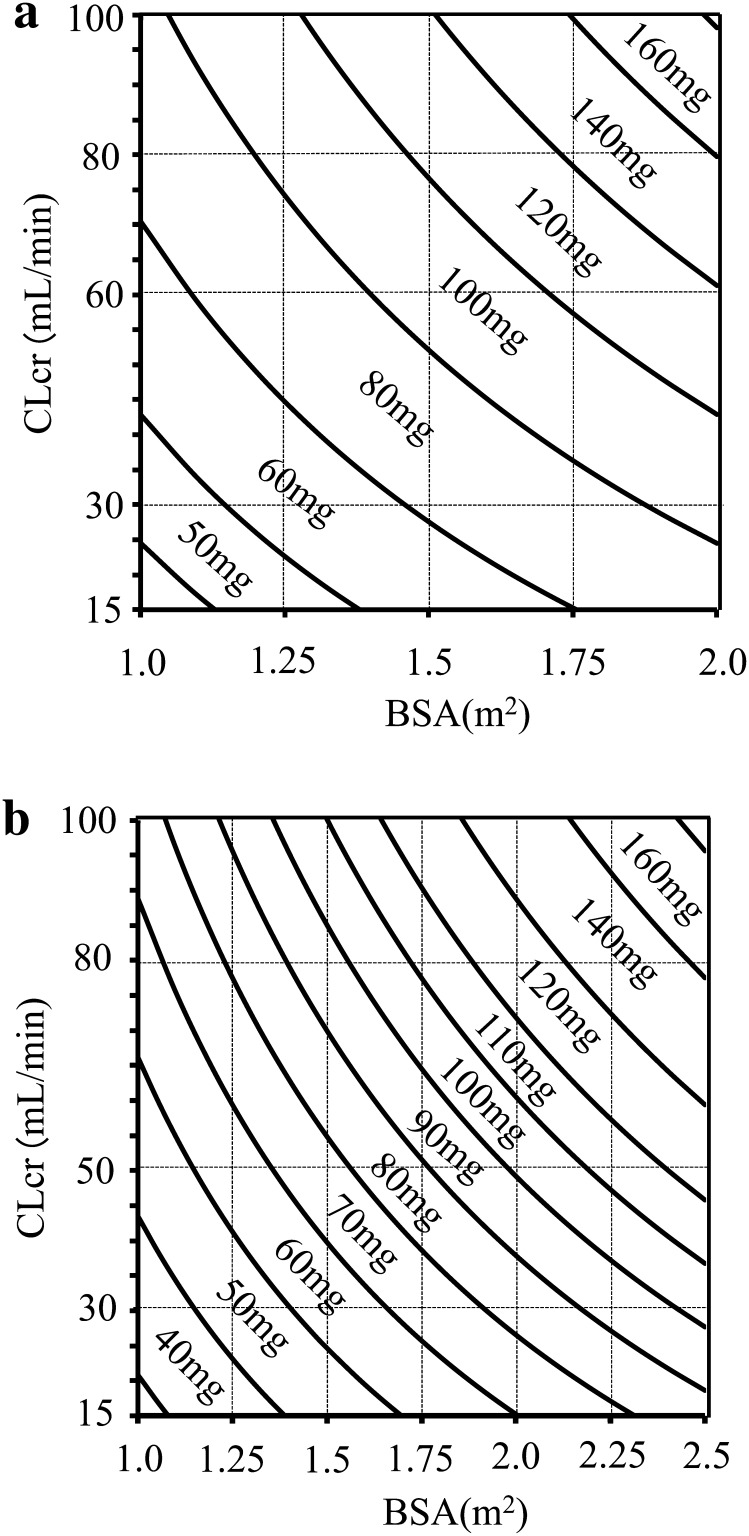
Recommended total daily doses of S-1 as tegafur, according to creatinine clearance (*CLcr*) estimated by the Cockcroft–Gault equation and body surface area (*BSA*) in Asia for the approved dose of 80 mg/m^2^ as tegafur (**a**) and in Europe for the approved dose of 50 mg/m^2^ as tegafur (**b**)

The target AUC of 5-FU was derived from previous reports of pivotal registration studies that confirmed the efficacy and toxicity of S-1 at the approved dose. The target AUCs of a single dose were defined as 723.9 and 588.6 ng h/mL in Asia (40 mg/m^2^, twice daily) [13] and Europe (25 mg/m^2^, twice daily) [22], respectively. The recommended daily doses in Asia and Europe calculated with Eq. 3 are shown as nomograms in Fig. 5 for Asia and Europe with consideration of each approved dosage (20 and 25 mg as tegafur in Asia, 15 and 20 mg as tegafur in Europe).

## Discussion

In our study, it was confirmed that renal dysfunction caused high exposure of CDHP and 5-FU in patients with CLcr in the range 15.9–108.8 mL/min. Ikeda et al. [14] observed increasing exposure of CDHP and 5-FU in rabbits with three degrees of renal dysfunction induced by cisplatin compared with exposure in rabbits with normal renal function. There have been some reports on the relationships between renal function and the AUC of CDHP and/or that of 5-FU in patients with cancer [14, 20, 21]; however, these studies did not include patients with severe renal impairment (CLcr ≥ 36.3  mL/min [14], CLcr ≥ 39.0 mL/min [20], and CLcr ≥ 54.0 mL/min [21]). Therefore, this is the first report to clarify the effect of renal function on the exposure of CDHP and 5-FU in patients with wide variation in renal function, including severe impairment (CLcr = 29.3 and 15.9 mL/min).

In patients with CLcr in the range 15.9–108.8 mL/min, the AUCs of both CDHP and 5-FU were correlated with CLcr and GFR (Fig. 4). Although GFR is widely used to assess renal function for diagnosing kidney disease, it is not always an appropriate measure of renal function for predicting drug exposure and adjusting the drug dosage in patients with renal impairment. Renal drug elimination comprises the combined processes of glomerular filtration, tubular secretion, and reabsorption. Putt et al. [23] demonstrated that measured GFR approximated renal tubular anion excretion and reabsorption, but did not correlate well with cationic drug transport. Furthermore, they suggested that dose adjustments based on GFR may underestimate clearances and potentially mislead the clinician to prescribe ineffective doses of important drugs eliminated via tubular pathways. This finding is very important for future discussion of our study, although the route of CDHP renal elimination remains unclear.

An S-1 dosage formula based on renal function as indicated by CLcr was derived according to the concept of exposure matching to the approved dose of S-1 in pivotal registration studies (Eq. 3). Systemic exposure is one of the indirect pharmacokinetic measures for product quality bioequivalence [24, 25]. The US Food and Drug Administration and the European Medicines Agency also recommend dose adjustment to produce a range of plasma concentrations of drugs or active metabolites that is similar in subjects with normal renal function and those with impaired renal function [1, 2]. Our S-1 dosage formula was developed by PPK analysis in 16 patients with various degrees of CLcr in the range 15.9–108.8 mL/min. The number of patients (16) is not insufficient to derive the initial formula by reference to the development of a carboplatin dosage formula by Calvert et al. [26]. They derived the initial formula from a retrospective analysis of carboplatin pharmacokinetics in only 18 patients with GFR in the range 33–136 mL/min, and evaluated it in 31 patients prospectively. The reliability of the formula we have developed must be evaluated subsequently for practical application.

We have proposed an original formula (Eq. 3) and nomograms (Fig. 5) illustrating the recommended dose of S-1 according to patients’ CLcr and BSA in consideration of the dosage (20 and 25 mg as tegafur in Asia, 15 and 20 mg as tegafur in Europe). On the Asian package insert, the dosage and administration are presented only for patients with normal renal function [7, 8]. Therefore, this is the first information on dose optimization for patients with impaired renal function in Asia. Furthermore, regarding dose modification, the European summary of product characteristics indicates that the standard dose should be administered without modification in patients with mild renal impairment (CLcr = 51–80 mL/min), whereas the drug should be administered at 40 mg/m^2^/day as tegafur for patients with moderate renal impairment (CLcr = 30–50 mL/min) [9]. These values were derived from a Monte Carlo simulation of virtual patients with renal dysfunction using a previously developed PPK model for S-1 in Western patients [27]. The limited guidance on S-1 dose adjustment in the summary of product characteristics is similar to the recommended dose provided by the formula we have developed. For patients with severe renal impairment, our formula also provides a novel dosing recommendation for S-1 in Europe.

Tegafur is a prodrug of 5-FU with good oral bioavailability. Following oral administration, tegafur is gradually converted to 5-FU, mainly by cytochrome P450 (CYP) 2A6 [28, 29]. Although CYP2A6 variants are associated with the pharmacokinetic variability of tegafur [30], the AUC of CDHP, which is affected by renal function, is the key determinant of the pharmacokinetic variability of 5-FU [20]. Generally, prior gastrectomy causes a change in the absorption of orally administered drugs. In our 16 patients, including nine patients who had previously undergone gastrectomy, a history of gastrectomy did not affect the individual pharmacokinetics of tegafur and CDHP according to the PPK analysis. The resulting exposure of 5-FU was not influenced by gastrectomy. The effect of gastrectomy on the pharmacokinetics of 5-FU after S-1 administration is unclear [19, 31–33]. However, as approximately only 10 % of absorbed tegafur is converted to 5-FU in terms of the plasma concentrations of tegafur and 5-FU, we do not consider a history of gastrectomy to be a crucial factor affecting the pharmacokinetics of 5-FU. Therefore, the formula we have developed is expected to apply for patients regardless of CYP2A6 polymorphism, which causes ethnic differences according to race-related differences in the allele frequency, and a history of gastrectomy.

In conclusion, we have developed a simple formula for determining the S-1 dosage on the basis of individual CLcr and BSA values. The recommended daily doses of S-1 in Asia and Europe were also proposed as nomograms according to each approved dose and dosage. Further validation is needed to confirm the formula for practical application.

## Electronic supplementary material

Below is the link to the electronic supplementary material.

Supplementary material 1 (PDF 63 kb)

Supplementary material 2 (PDF 70 kb)

## References

[1] US Food Drug Administration. Guidance for industry: pharmacokinetics in patients with impaired renal function—study design, data analysis, and impact on dosing and labeling. 2010. http://www.fda.gov/downloads/Drugs/GuidanceComplianceRegulatoryInformation/Guidances/ucm072127.pdf. Accessed 21 July 2015.

[2] European Medicines Agency. Note for guidance on the evaluation of the pharmacokinetics of medical products in patients with renal function. 2004. http://www.ema.europa.eu/docs/en_GB/document_library/Scientific_guideline/2009/09/WC500003123.pdf. Accessed 21 July 2015.

[3] Heidelberger C, Chaudhuri NK, Danneberg P, Mooren D, Griesbach L, Duschinsky R (1957). Fluorinated pyrimidines, a new class of tumour-inhibitory compounds. Nature.

[4] Shirasaka T, Nakano K, Takechi T, Satake H, Uchida J, Fujioka A (1996). Antitumor activity of 1 M tegafur-0.4 M 5-chloro-2,4-dihydroxypyridine-1 M potassium oxonate (S-1) against human colon carcinoma orthotopically implanted into nude rats. Cancer Res.

[5] Shirasaka T, Shimamato Y, Ohshimo H, Yamaguchi M, Kato T, Yonekura K (1996). Development of a novel form of an oral 5-fluorouracil derivative (S-1) directed to the potentiation of the tumor selective cytotoxicity of 5-fluorouracil by two biochemical modulators. Anticancer Drugs.

[6] Shirasaka T, Shimamoto Y, Fukushima M (1993). Inhibition by oxonic acid of gastrointestinal toxicity of 5-fluorouracil without loss of its antitumor activity in rats. Cancer Res.

[7] Taiho Pharmaceutical. TS-1 prescribing information in Japan; TS-1 combination OD tablet. Ver. 4; July 2014.

[8] Taiho Pharmaceutical. TS-1 prescribing Information in Singapore. Revised: TS-ONE capsule; 2013.

[9] European Medicines Agency. Teysuno : EPAR - product information. 2013. http://www.ema.europa.eu/docs/en_GB/document_library/EPAR_-_Product_Information/human/001242/WC500104415.pdf. Accessed 21 July 2015.

[10] Sakuramoto S, Sasako M, Yamaguchi T, Kinoshita T, Fujii M, Nashimoto A (2007). Adjuvant chemotherapy for gastric cancer with S-1, an oral fluoropyrimidine. N Engl J Med.

[11] Boku N, Yamamoto S, Fukuda H, Shirao K, Doi T, Sawaki A (2009). Fluorouracil versus combination of irinotecan plus cisplatin versus S-1 in metastatic gastric cancer: a randomised phase 3 study. Lancet Oncol.

[12] Koizumi W, Narahara H, Hara T, Takagane A, Akiya T, Takagi M (2008). S-1 plus cisplatin versus S-1 alone for first-line treatment of advanced gastric cancer (SPIRITS trial): a phase III trial. Lancet Oncol.

[13] Hirata K, Horikoshi N, Aiba K, Okazaki M, Denno R, Sasaki K (1999). Pharmacokinetic study of S-1, a novel oral fluorouracil antitumor drug. Clin Cancer Res.

[14] Ikeda M, Furukawa H, Imamura H, Shimizu J, Ishida H, Masutani S (2002). Pharmacokinetic study of S-1, a novel oral fluorouracil antitumor agent in animal model and in patients with impaired renal function. Cancer Chemother Pharmacol.

[15] Cockcroft DW, Gault MH (1976). Prediction of creatinine clearance from serum creatinine. Nephron.

[16] US Department of Health and Human Services. Common Terminology Criteria for Adverse Events (CTCAE) version 4.0. 2010. http://evs.nci.nih.gov/ftp1/CTCAE/CTCAE_4.03_2010-06-14_QuickReference_5x7.pdf. Accessed 21 July 2015.

[17] Matsuo S, Imai E, Horio M, Yasuda Y, Tomita K, Nitta K (2009). Revised equations for estimated GFR from serum creatinine in Japan. Am J Kidney Dis.

[18] Liu K, Zhong D, Zou H, Chen X (2010). Determination of tegafur, 5-fluorouracil, gimeracil and oxonic acid in human plasma using liquid chromatography-tandem mass spectrometry. J Pharm Biomed Anal.

[19] Hirose T, Fujita K, Nishimura K, Ishida H, Yamashita K, Sunakawa Y (2010). Pharmacokinetics of S-1 and CYP2A6 genotype in Japanese patients with advanced cancer. Oncol Rep.

[20] Fujita K, Yamamoto W, Endo S, Endo H, Nagashima F, Ichikawa W (2008). CYP2A6 and the plasma level of 5-chloro-2,4-dihydroxypyridine are determinants of the pharmacokinetic variability of tegafur and 5-fluorouracil, respectively, in Japanese patients with cancer given S-1. Cancer Sci.

[21] Ando Y, Kawada K, Inada M, Morita S, Mitsuma A, Yasuda Y (2012). Pharmacokinetic study of S-1 in patients in whom inulin clearance was measured. Oncology.

[22] van Groeningen CJ, Peters GJ, Schornagel JH, Gall H, Noordhuis P, de Vries MJ (2000). Phase I clinical and pharmacokinetic study of oral S-1 in patients with advanced solid tumors. J Clin Oncol.

[23] Putt TL, Duffull SB, Schollum JB, Walker RJ (2014). Gfr may not accurately predict aspects of proximal tubule drug handling. Eur J Clin Pharmacol.

[24] US Food Drug Administration. Guidance for industry: bioavailability and bioequivalence studies for orally administered drug products - general considerations. 2003. http://www.fda.gov/downloads/Drugs/Guidances/ucm070124.pdf. Accessed 21 July 2015.

[25] European Medicines Agency. Guideline on the investigation of bioequivalence. 2010. http://www.ema.europa.eu/docs/en_GB/document_library/Scientific_guideline/2010/01/WC500070039.pdf. Accessed 21 July 2015. 10.1111/j.1742-7843.2009.00518.x 20070293

[26] Calvert AH, Newell DR, Gumbrell LA, O’Reilly S, Burnell M, Boxall FE (1989). Carboplatin dosage: prospective evaluation of a simple formula based on renal function. J Clin Oncol.

[27] Comets E, Ikeda K, Hoff P, Fumoleau P, Wanders J, Tanigawara Y (2003). Comparison of the pharmacokinetics of S-1, an oral anticancer agent, in Western and Japanese patients. J Pharmacokinet Pharmacodyn.

[28] Ikeda K, Yoshisue K, Matsushima E, Nagayama S, Kobayashi K, Tyson CA (2000). Bioactivation of tegafur to 5-fluorouracil is catalyzed by cytochrome P-450 2A6 in human liver microsomes in vitro. Clin Cancer Res.

[29] Komatsu T, Yamazaki H, Shimada N, Nakajima M, Yokoi T (2000). Roles of cytochromes P450 1A2, 2A6, and 2C8 in 5-fluorouracil formation from tegafur, an anticancer prodrug, in human liver microsomes. Drug Metab Dispos.

[30] Ichikawa W, Fujita K, Sasaki Y (2008). The unanswered question: what is the determinant of S-1 pharmacokinetics?. Clin Pharmacol Ther.

[31] Kim WY, Nakata B, Hirakawa K (2007). Alternative pharmacokinetics of S-1 components, 5-fluorouracil, dihydrofluorouracil and alpha-fluoro-beta-alanine after oral administration of S-1 following total gastrectomy. Cancer Sci.

[32] Kochi M, Fujii M, Kanamori N, Kaiga T, Aizaki K, Takahashi T (2007). Effect of gastrectomy on the pharmacokinetics of S-1, an oral fluoropyrimidine, in resectable gastric cancer patients. Cancer Chemother Pharmacol.

[33] Tsuruoka Y, Kamano T, Kitajima M, Kawai K, Watabe S, Ochiai T (2006). Effect of gastrectomy on the pharmacokinetics of 5-fluorouracil and gimeracil after oral administration of S-1. Anticancer Drugs.

