# Supplementary material for: Development of an S-1 dosage formula based on renal function by a prospective pharmacokinetic study
Source: Gastric Cancer. 2015 Aug 25;19:876–86. doi: 10.1007/s10120-015-0536-6 (PMC4906077; doi:10.1007/s10120-015-0536-6)
Supplement: Supplementary file 1 — Supplementary material 1 (PDF 63 kb) [file 10120_2015_536_MOESM1_ESM.pdf]

Fig. S1

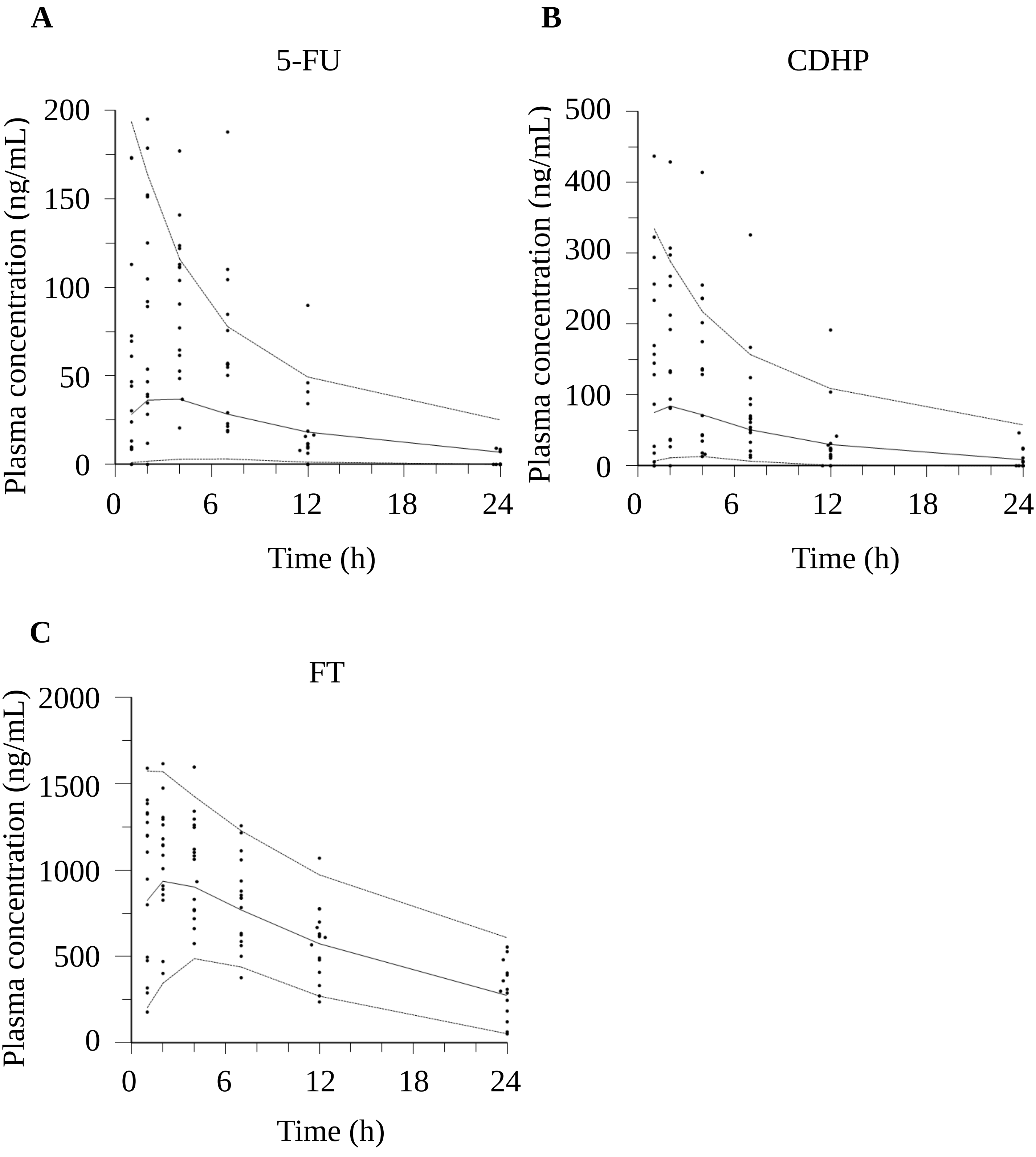
A
5-FU
Plasma concentration (ng/mL)
200
150
100
50
0
0
6
12
18
24
Time (h)
B
CDHP
Plasma concentration (ng/mL)
500
400
300
200
100
0
0
6
12
18
24
Time (h)
C
FT
Plasma concentration (ng/mL)
2000
1500
1000
500
0
0
6
12
18
24
Time (h)

Supplementary figure legend

Figure S1. Simulated plasma concentration–time profiles of 5-FU (A), CDHP (B), and FT (C) after a single oral dose of S-1 (40 mg/m^2 as FT).

Plotted symbols show the observed concentration data and the lines represent the results of visual predictive checks. The 5th, 50th, and 95th percentiles (from bottom to top) of the simulated concentrations (1000 replications) are displayed using the final population PK model presented in Table 2.
