# Supplementary material for: Development of an S-1 dosage formula based on renal function by a prospective pharmacokinetic study
Source: Gastric Cancer. 2015 Aug 25;19:876–86. doi: 10.1007/s10120-015-0536-6 (PMC4906077; doi:10.1007/s10120-015-0536-6)
Supplement: Supplementary file 2 — Supplementary material 2 (PDF 70 kb) [file 10120_2015_536_MOESM2_ESM.pdf]

Table S1. Bootstrap validations for the final estimates of population pharmacokinetic parameters of 5-FU, CDHP, and FT

Compound	Parameters	Final results by original dataset		Bootstrap re-sampling (n=1000)	
		Estimates	S.E.	Median	95% Confidence Interval
5-FU	Ka (h^{-1})	0.551	0.0499	0.547	0.436–0.687
	V/F (L/m^2)	362	45.5	346	251–478
	CL/F ($L/h/m^2$) = THETA(1) + THETA(2) × CLcr				
	THETA(1)	21.9	3.28	22.5	10.3–51.6
	THETA(2)	0.375	0.0775	0.361	1.26E-05–1.42
	ω^2 (Ka)	4.09	1.66	3.74	0.997–7.93
	ω^2 (V/F)	0.768	0.504	0.640	0.0904–2.22
	ω^2 (CL/F)	0.0759	0.0267	0.0671	0.0173–0.123
CDHP	Ka (h^{-1})	1.04	0.252	1.01	0.577–1.66
	V/F (L/m^2)	200	29.7	191	137–279
	CL/F ($L/h/m^2$) = THETA(1) + THETA(2) × CLcr				
	THETA(1)	3.77	1.65	4.01	0.0143–28.3
	THETA(2)	0.403	0.0873	0.383	0.00726–154
	ω^2 (Ka)	2.78	0.862	2.71	1.03–5.64
	ω^2 (V/F)	0.624	0.334	0.507	0.00165–1.84
	ω^2 (CL/F)	0.249	0.0845	0.234	0.0463–0.611
FT	Ka (h^{-1})	1.46	0.234	1.49	1.18–2.41
	V/F (L)	44.9	3.01	45.3	40.1–51.7
	CL/F (L/h)	2.89	0.350	2.89	2.30–3.66
	ω^2 (Ka)	1.46	0.319	1.56	0.959–2.87
	ω^2 (V/F)	0.0558	0.0214	0.0496	0.0099–0.0955
	ω^2 (CL/F)	0.175	0.0775	0.155	0.055–0.369

Abbreviations: S.E., standard error; Ka, first-order absorption rate constant; V, Volume of distribution; F, bioavailability; CL, clearance; CLcr, creatinine clearance estimated by the Cockcroft–Gault equation.
